# Systematic evaluating and modeling of SARS-CoV-2 UVC disinfection

**DOI:** 10.1038/s41598-022-09930-2

**Published:** 2022-04-07

**Authors:** Sebastian Freeman, Karen Kibler, Zachary Lipsky, Sha Jin, Guy K. German, Kaiming Ye

**Affiliations:** 1 Department of Biomedical Engineering, Binghamton University, State University of New York (SUNY), PO Box 6000, Binghamton, NY 13902 USA; 2 Center of Biomanufacturing for Regenerative Medicine, Binghamton University, State University of New York (SUNY), Binghamton, NY 13902 USA; 3 Biodesign Institute, Arizona State University, McAllister Ave, Tempe, AZ 85281 USA

**Keywords:** Biotechnology, Microbiology, Health care

## Abstract

The ongoing COVID-19 global pandemic has necessitated evaluating various disinfection technologies for reducing viral transmission in public settings. Ultraviolet (UV) radiation can inactivate pathogens and viruses but more insight is needed into the performance of different UV wavelengths and their applications. We observed greater than a 3-log reduction of SARS-CoV-2 infectivity with a dose of 12.5 mJ/cm^2^ of 254 nm UV light when the viruses were suspended in PBS, while a dose of 25 mJ/cm^2^ was necessary to achieve a similar reduction when they were in an EMEM culture medium containing 2%(v/v) FBS, highlighting the critical effect of media in which the virus is suspended, given that SARS-CoV-2 is always aerosolized when airborne or deposited on a surface. It was found that SARS-CoV-2 susceptibility (a measure of the effectiveness of the UV light) in a buffer such as PBS was 4.4-fold greater than that in a cell culture medium. Furthermore, we discovered the attenuation of UVC disinfection by amino acids, vitamins, and niacinamide, highlighting the importance of determining UVC dosages under a condition close to aerosols that wrap the viruses. We developed a disinfection model to determine the effect of the environment on UVC effectiveness with three different wavelengths, 222 nm, 254 nm, and 265 nm. An inverse correlation between the liquid absorbance and the viral susceptibility was observed. We found that 222 nm light was most effective at reducing viral infectivity in low absorbing liquids such as PBS, whereas 265 nm light was most effective in high absorbing liquids such as cell culture medium. Viral susceptibility was further decreased in N95 masks with 222 nm light being the most effective. The safety of 222 nm was also studied. We detected changes to the mechanical properties of the stratum corneum of human skins when the 222 nm accumulative exposure exceeded 50 J/cm^2^.The findings highlight the need to evaluate each UV for a given application, as well as limiting the dose to the lowest dose necessary to avoid unnecessary exposure to the public.

## Introduction

While vaccines provide an effective protection from SARS-CoV-2 infection, the efficiency of these vaccines against emerging SARS-CoV-2 variants is largely unknown^1^. Globally, we are still facing significant challenges in controlling COVID. Evaluating additional strategies for reducing public health risks are of importance not only now, but also for improving future pandemic responses, in particular during periods prior to vaccine development for new and emergent pathogens.

A number of disinfection technologies have been explored to eliminate SARS-CoV-2 from contaminated surfaces or in the air. SARS-CoV-2 is an enveloped virus. It is generally recognized as being highly susceptible to most cleaning agents^2^ and alcohol-based hand sanitizing solutions^3^. This approach provides effective disinfection but must be applied thoroughly over all surfaces, and generally by humans. Automated systems such as spray curtains can improve the efficiency^4–6^ but are not practical for most disinfection applications in the spaces other than in laboratories or healthcare settings.

There is increased interest in developing antimicrobial surface coatings for rapid and sustained disinfection^7–10^. For instance, quaternary ammonium, a well-tested antimicrobial agent, when combined with organosilanes, can achieve a microbial reduction of > 99.9999% even after 24 h after its application^9^. The major disadvantage of these antimicrobial coating approaches is that their effectiveness can be affected by many unpredicted factors. For instance, the quaternary ammonium will lose its effectiveness when mixed with organic matter such as the presence of soil, blood, etc. Also, these agents are lung irritants and can contribute to asthma and other breathing problems.

Lower wavelength between 200 and 290 nm ultraviolet light (UVC) causes DNA and RNA damage, primarily through the mechanism of thymine and pyrimidine dimers, which disrupt nucleic acid replications and therefore inactivate various pathogens, including viruses such as SARS-CoV-2^11–16^. Consequentially, UVC-based disinfection systems have become increasingly visible in the public sphere as a reliable method of disinfection, and UVC-based disinfection is a promising tool to reduce SARS-CoV-2 transmission due to its low cost and manageable risk^17^. Measures that reduce transmission rates in public settings are critical to reopening the economy and bringing everyday life back to normal, particularly for in-door activities. An effective UVC disinfection system can eradicate both airborne viruses and contaminated surfaces, helping to prevent human–human transmission. This will enable the restoration of many in-door activities and allow people to return to work before a vaccine is available.

There are three UVC range subsets that have been extensively studied as optimal for pathogen disinfection^18,19^: (1) 207–222 nm^13,20–22^, (2) 254 nm^23–26^, and (3) 260–280 nm^24,27–30^. 254 nm wavelength is the most commonly used germicidal wavelength. However, the 254 nm wavelength can damage skin and eyes^31–33^. As such, disinfection methods utilizing 254 nm wavelength UV light must be employed only in unoccupied spaces, or must be designed so as to not cause direct exposure. Wavelengths in the 260–280 nm range have been purported to be more efficient than 254 nm, as these wavelengths are closer to the maximum absorption of the RNA or DNA ^34,35^. Furthermore, wavelengths of 207–222 nm have gained an increasing spotlight as a novel disinfection wavelength. Such wavelengths minimally reach the earth’s surface due to attenuation by atmospheric ozone. Recent reports have shown a high germicidal effectiveness of 222 nm wavelength light^13,15,21,36^. Studies also suggested that 222 nm UVC light may be safely used in public settings, as it cannot penetrate tissue as deeply as a higher wavelength UVC can^21,37^.

Further complicating the evaluation of the different UVC wavelengths is the widely varying inactivation doses reported to achieve a certain log-reduction for various pathogens. A broad review of coronaviruses and their inactivation doses revealed a wide range of recommended doses from 0.6 to 11,754 mJ/cm^2^ ^34^. Such inconsistencies might be caused by varied experimental conditions and setups employed as a potential confounding factor in the determination of the necessary dose. It highlights a substantial need to standardize a testing platform in order to evaluate germicidal inactivation doses while at the same time to understand how environmental factor influence the inactivation doses for a given pathogen. This is critical not only to the determination of UVC inactivation doses but also to the regulation of UVC products on the market. Un- or ill-regulated UVC products will jeopardize our ability to mitigate the COVID pandemic and also expose the public to risks due to their false safety assurance.

Extended UV exposure is associated with increased skin aging/degradation ^40,41^, sunburn^42^, and an increased propensity to develop skin melanoma^43^. These UV-induced effects can be caused directly by the formation of cyclobutene pyrimidine dimers or photoproducts^42,44–47^, or indirectly through the production of reactive oxygen species, which damage DNA, proteins, and lipids through oxidative stress^38,42,48^. The wavelength and exposure dosage of UV light can also influence the method and level of skin damage^45,49^.

The pandemic put a great strain on the supply and availability of various personal protective equipment (PPE) such as N95 respirators. Although it is designed for single use and disposable, the desperate situation made it relevant to evaluate the potential reuse after proper disinfection. Although 254 nm has been evaluated and compared to other methods^6,16,25,48,49^, other wavelengths such as LED UVC with relatively high wavelengths that can be used safely were less studied.

Herein, we developed a UVC disinfection dose determination model and used it to determine how required doses changed in different experimental conditions and demonstrate how the effective dose required to completely eradicate SARS-CoV-2 through UVC irradiation changes depending on experimental conditions. The discovery of the media effect on viral UVC disinfection highlights the need for laboratories to use relevant media when performing UVC disinfection dose determination that will influence public health recommendations. Considerations such as these should be used by industries when designing their UVC products and by agencies when regulating the UVC products designed for disinfecting SARS-CoV-2. Using this model, we shed some light on the disparate disinfection doses recommended in the literature for SARS-CoV-2. Our study supports the idea that the effectiveness of UVC disinfection is dependent upon wavelength, energy, distance, exposure time, and the media type and volume where viruses are suspended or enclosed. In addition, we report on important safety considerations when implementing UVC light for disinfection, particular with respect to UVC skin safety, as well as its potential effectiveness for the disinfection of N95 respirators.

## Materials and methods

### UVC dose determination

**Figure 1:**
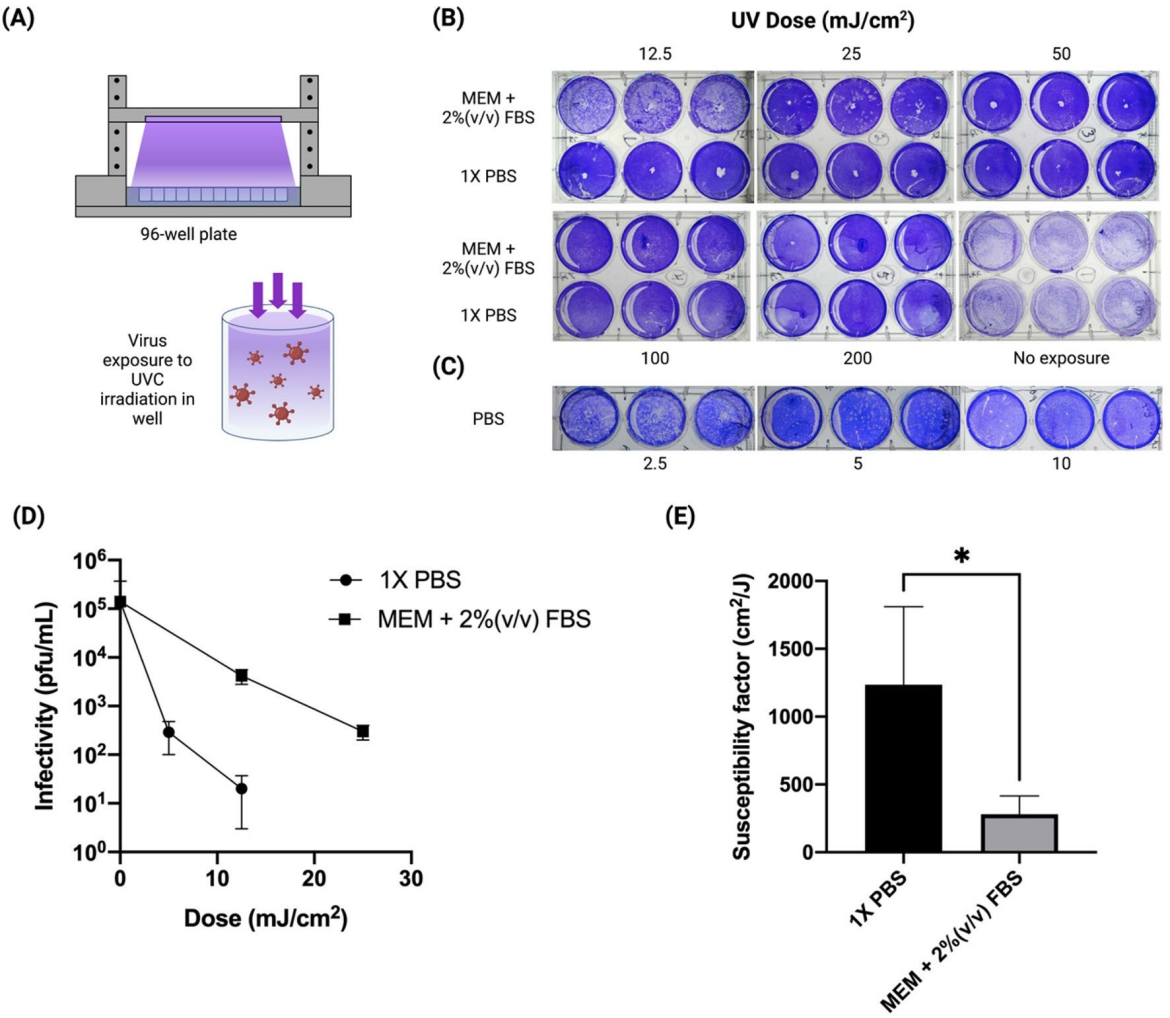
Inactivation of SARS-CoV-2 by UVC irradiation. (**A**) A schematic diagram of the UVC dose testing device. (**B**) A plaque-forming assay of SARS-CoV-2 infectivity after a high dose (12.5–200 mJ/cm^2^) UVC treatment. (**C**) A plaque-forming assay of SARS-CoV-2 infectivity after a low dose (2.5–10 mJ/cm^2^) UVC treatment performed in 1X PBS. Controls were treated exactly the same, but did not receive any UV light exposure. (**D**) UVC 254 nm inactivation curves from the plaque-forming assay for SARS-CoV-2 in 1 × PBS or MEM + 2%(v/v) FBS. (**E**) Susceptibility factors calculated from the UVC inactivation (*p value < 0.05).

To quantify the UVC dose, a UVC radiometer (International Light Technologies, MA) was used to map the irradiance under each light source. We used a low-pressure handheld 254 nm mercury lamp (UVS-24, Analytik-Jena), a 222 nm excimer lamp (Care222, USHIO), and an LED array consisting of UVC 265 nm 60 mW LED (KL265, Klaran Crystal IS). All light sources were warmed up for at least 10 min prior to use and were kept cool by a fan to keep the output intensity consistent. A custom-built aluminum fixture (Fig. 1A) was built to support the light source over a 96-well plate. The area under the light source was measured at evenly-spaced intervals in a 6 × 6 rectangular grid. This process was repeated for at least four different height levels. The gridded irradiance data was fed into MATLAB by linear interpolation to predict values between our measured points in the XY plane, and a cubic interpolation to predict values between our measured points in the Z direction. The average irradiance was calculated for each well of the plate. Exposure times were calculated using Eq. (1),

1 $$t = \frac{D}{{E_{avg} }}$$

where $t$ is the exposure time, $D$ is the desired exposure dose, and $E_{avg}$ is the average irradiance for a given well.

### SARS-CoV-2 and lentiviral UVC dose titration

SARS-CoV-2, isolate USA-WA1/2020 (BEI resources # NR52281, a gift from Dr. Mark Mulligan at the NYU Langone Vaccine Center) was used for our study. A portion of 300 µL of 10^6^ pfu/mL SARS-CoV-2 was suspended in either PBS (Mediatech, 21-040CV, VA) or Eagle’s minimum essential medium (EMEM) (ATCC, 30-2003, VA) medium supplemented with 2%(v/v) FBS (HyClone Laboratories, SH30396, UT) in a 96-well plate placed on the platform and exposed to various doses of UVC 254 nm light (Analytik Jena, CA) (2.5 mJ/cm^2^–1 J/cm^2^). Following UVC treatment, all virus samples were immediately frozen and later thawed to quantify by a plaque-forming assay in African green monkey Vero E6 cells (ATCC CRL-1586). Vero E6 cells are a susceptible cell line for SARS-CoV-2 infection and were maintained during the infection in the aforementioned EMEM medium supplemented with 2%(v/v) FBS. The thawed suspension of 10^6^ pfu/mL of SARS-CoV-2 was added to a confluent cell monolayer for an infection period of 1 h in a CO_2_ incubator at 37 °C. Following the infection incubation, the monolayers were overlaid with 0.8% final concentration of agarose in 2 × MEM (Thermo Fisher-Gibco, 11935046, MA) supplemented with 4% FBS. The infected cells were incubated for 48 h; agarose overlays were removed and the cells stained with crystal violet solution (20% EtOH and 0.2% w/v crystal violet, Fisher C581-100, MA) to reveal plaques. Conditions were performed in triplicate and plaques were manually counted to determine the log reduction of virus infectivity after UVC treatment.

The lentivirus used for UVC dose titration was pLenti CMV GFP Hygro (654-4), a gift from Eric Campeau & Paul Kaufman^50^ (Addgene viral prep #17446-LV). The cells used for titration were human embryonic kidney cell line HEK293-T cells (ATCC CRL-1128). HEK293T cells were maintained in a high glucose DMEM (Gibco, 11995) containing 2 mM L-glutamine, 1 mM sodium pyruvate, and supplemented with 1000 U/mL penicillin, 1 mg/mL streptomycin, and 10% heat-inactivated FBS (Gibco, 10437010). HEK293-T cell passages up to 15 were used for the experiment. For UVC dose experiments, the viruses were exposed to various doses of UVC and infectivity of the viruses after UVC treatment was determined by infecting HEK293-T cells with the treated viruses. 96-well plates containing viral liquid suspensions were placed directly below the UV light source at a distance approximate 3 cm from the liquid surface. In brief, 3X10^3^ HEK293-T cells/cm^2^ were seeded in 48-well plates and incubated at 37 °C and 5% CO_2_ overnight in the aforementioned medium supplemented with 20 mM HEPES to protect from pH changes while plates were processed. A separate plate of HEK 293-T cells was prepared using the same seeding conditions and was used to estimate cell count at Day 0. The cells were infected with UVC-treated viruses the next day to determine log reduction in infectivity of the UVC treated viruses. A multiplicity of infection (MOI) of 10 was used for infection. UVC treatment was performed in the wells of a polystyrene 96-well plate. After exposure, the treated viral suspensions were combined 1:1 (v:v) with a solution of 40 mM HEPES + 16 µg/mL polybrene in a complete cell culture medium (termed transduction medium) and used to infect HEK 293 T cells. The cells were incubated with 100 µL of the virus containing transduction medium at 37 °C and 5% CO_2_ for 2 h. Afterwards, an additional 100 µL of the complete cell culture medium + 20 mM HEPES were added to the 48-well plate cell culture wells before placing them in the incubator for two days to allow for GFP expression. The GFP expression in the infected cells was confirmed using an inverted fluorescent microscope (Nikon TI Eclipse).

### Infectivity assay

A high-content Cytation 5 imaging reader (Biotek, Winooski, VT) was used to take tiled 6 × 6 fluorescent images or larger of each 48-well plate with a 10 × objective lens. Virus infected GFP expressing HEK 293 T cells were counterstained with 1:2000 diluted Hoechst 33,342 for 20 min before imaging. Similarly, HEK293T cells were incubated with Hoechst 33,342 and imaged at Day 0 (the day transduction took place) to estimate cell count. A custom MATLAB script was written to quantify nuclei and the number of GFP expressing cells in order to determine the percentage of fluorescent cells in each well. The active GFP-lentivirus concentration (TU/mL) was calculated using Eq. (2),

2 $$C = \frac{{ - \ln \left( {1 - p} \right)*\overline{c}}}{V}$$

where $C$ is the active virus concentration, $p$ is the percentage of GFP-expressing cells, $\overline{c}$ is the average number of cells counted on the day the transduction took place, and $V$ is the volume of lentiviral suspension added to each well during UVC exposure.

### N95 respirator sample testing

7-mm diameter cut-out samples were cut out of a respirator mask (Model 8511, 3 M, Saint Paul, MN). The respirator masked used in this study consisted of three distinct layers. A sterile hole puncher was used to cut the mask samples to uniform a uniform shape. Distinct layers were carefully manipulated to keep original organization intact using sterile tweezer and allocated into the wells of a 96-well plate in preparation to the experiment with the external surface facing up. 10 µL of a 7 × 10^5^ TU virus containing solution were wetted onto the respirator cut-out samples and allowed to dry for 20 min prior to UV disinfection. Cut-out samples were placed into the wells of a 96-well plate with the external mask surface facing up. After exposure, a solution of cell culture medium supplemented with 20 mM HEPES and 8 µg/mL polybrene and incubated at room temperature for 20 min. 100 µL of recovered solution was employed to infect HEK 293-T cells as described above. Titer analysis was performed as described above.

### UVC absorbance measurement

A Cytation 5 imaging reader (Biotek, Winooski, VT) was used to measure the UV absorbance spectra of different liquids. For all absorbance measurements, liquids were aliquoted into the wells of a UV-transparent 96-well plates (Corning Life Sciences, Corning, NY). Attenuation coefficients were calculated by aliquoting solution volumes of various heights in triplicate. Optical pathlength and absorbance plate reader measurements were performed at 230, 254, and 265 nm. Our plate reader lower wavelength limit of 230 nm was used as a substitute for measuring at 222 nm. The attenuation coefficient is the slope of the linear regression of absorbance versus optical pathlength and was reported for each vitamin and amino acid component of DMEM formulation (Gibco, 11,995) at their formulated concentrations.

### Skin damage analysis

Full thickness 26 years old female breast skin was obtained from the Yale Pathology Tissue Services (New Haven, CT) within 24 h of elective surgery. An exempt approval (3002–13) was obtained from the Binghamton University’s Institutional Review Board (IRB) to perform research using de-identified tissue samples pursuant to the Department of Health and Human Services (DHHS) regulations, 45 CFR 46.101:b:4. Breast tissue was used due to its low level of typical solar UV exposure. All methods were performed in accordance with the relevant guidelines and regulations. Stratum corneum (SC) was isolated from the epidermis using a standard heat bath and trypsin technique, placed on plastic mesh (Darice, Strongsville, OH), rinsed in deionized water, and dried for 48 h at room temperature and humidity. SC samples were cut to a uniform 0.95 × 1.9 cm using a rectangle punch. SC samples were exposed to 254 nm or 222 nm. SC samples exposed only to ambient UV light were used for controls. After UV irradiation, samples were equilibrated for 24 h to either 25% or 100% relative humidity (RH) using an airtight container filled with desiccant (Drierite 10-2 mesh, W.A. Hammond Drierite Company, Xenia, OH) or a hydration cabinet (F42072-1000, Secador, Wayne, NJ) with a base filled with deionized water, respectively. A hydrometer with probe (445815, Extech Instruments, Nashua, NH) was used to monitor RH in both cases throughout the equilibration period. After equilibration, samples were mechanically tested in a uniaxial tensometer (UStretch, CellScale, Waterloo, ON, Canada) equipped with a 4.4 N load cell and strained until rupture at a constant strain rate of 0.012 s^−1^; similar to rates used in previous mechanical studies of skin^51^. Tensile forces and grip separation were recorded at a frequency of 5 Hz. Optical microscopy using an Eclipse Ti-U inverted microscope (Nikon, Melville, NY) with 40 × oil objective lens was employed to determine the average thickness of ruptured SC samples. Optical thickness measurements were taken a distance from the crack interface to prevent measuring reduced thicknesses arising from plastic deformation. Sample dimension and recorded force displacement data were then used to derive engineering stress–strain curves, from which the average elastic modulus, fracture stress, fracture strain and work of fracture were determined ($4 \le n \le 5$ independent samples for every UV wavelength, dosage, and humidity).

### Statistical analysis

All statistical analysis was performed using R (version 3.4.2) and GraphPad Prism 9. For all lentiviral experiments, a one-way ANOVA was used to test for statistical significance, where the measured percentage of infected cells in the experimental conditions were compared to its respective control, which consisted of samples unexposed to UV light in the same liquid for the same amount of time. For skin experiments, a one-way ANOVA was used to test for statistical significance, where each UV condition (222 nm and 254 nm) was compared to the control across all dosages. Levene’s and Shapiro–Wilk’s tests were respectively used to determine equality of variances and normality. Post-hoc analyses were performed if statistical significance levels below 5% were established.

## Results

### Determining a UVC dose for eradicating SARS-CoV-2

UVC irradiation is known to be able to inactivate viruses including SARS-CoV-2. However, the required disinfection dose for SARS-CoV-2 remains unclear, despite its critical importance in the design and regulation of any UVC technology aimed at disinfecting SARS-CoV-2 during the COVID 19 pandemic. To ascertain the required dosage, a UVC irradiation testing device was designed and fabricated, as shown in Fig. 1A. UVC 254 nm light was used as the UV source, given that it is the most widely used UVC light for disinfection. The height between the UVC light and a loading platform used for placing the virus loaded plate can be adjusted in order to test different doses for disinfection. A UVC meter was used to measure the UVC irradiation. The dose (J/cm^2^) of UVC irradiation was calculated by multiplying the irradiation energy by the exposure time. The dose was altered by varying the exposure time and/or the height between the UVC light and the platform where the viruses were loaded. The Vero E6 cells were used to determine a log reduction of virus infectivity after exposure to UVC. We originally tested higher doses up to 1 J/cm^2^ but detected no plaques in doses as low as 0.3 J/cm^2^ (Fig. S1). We subsequently lowered the doses and observed a dose-dependent inactivation starting at 25 mJ/cm^2^ for conditions in MEM + 2%FBS, and 10 mJ/cm^2^ for PBS (Fig. 1A–C). Figure 1D shows a viral inactivation curve for SARS-CoV-2 determined from the plaque-forming assay. To determine the influence of environmental factors on UVC disinfection efficiency, we used susceptibility as a measure of UVC irradiation efficiency. Applying a single-phase exponential decay curve, the susceptibility, $k$ can be calculated using Eq. (3) as follow:

3 $$k = - \frac{\ln \left( s \right)}{D}$$

where $s$ is the viral survival fraction, and $D$ is the exposure dose necessary to achieve the fraction.

Applying the Eq. (2), we estimated a 254 nm susceptibility factor of 1,239 cm^2^/J in PBS and 281 cm^2^/J in MEM + 2%FBS, resulting in a 4.4-fold increase in susceptibility (Fig. 1E). Furthermore, these doses were considerably different from the ones reported in the literature, which vary widely.

This result raises an important question: what factor(s) plays a critical role in the UVC dosage required for viral inactivation? We hypothesized that the medium in which viruses are suspended attenuates UVC light, reducing the efficacy of UVC in killing viruses. The validation of this hypothesis is of paramount importance given that SARS-Cov-2 particles are always aerosolized during human-to-human transmission. The dose determined using lab-conditioned viruses, such as suspending viruses in a PBS or a cell culture medium, might not be accurate. A UVC device designed based on these doses might not be able to effectively eradicate SARS-CoV-2. To this end, we developed an experiment platform based on a GFP lentivirus to further explore these differences observed in our initial SARS-CoV-2 experiment.

### Effect of liquid suspension medium on virus UVC disinfection

**Figure 2:**
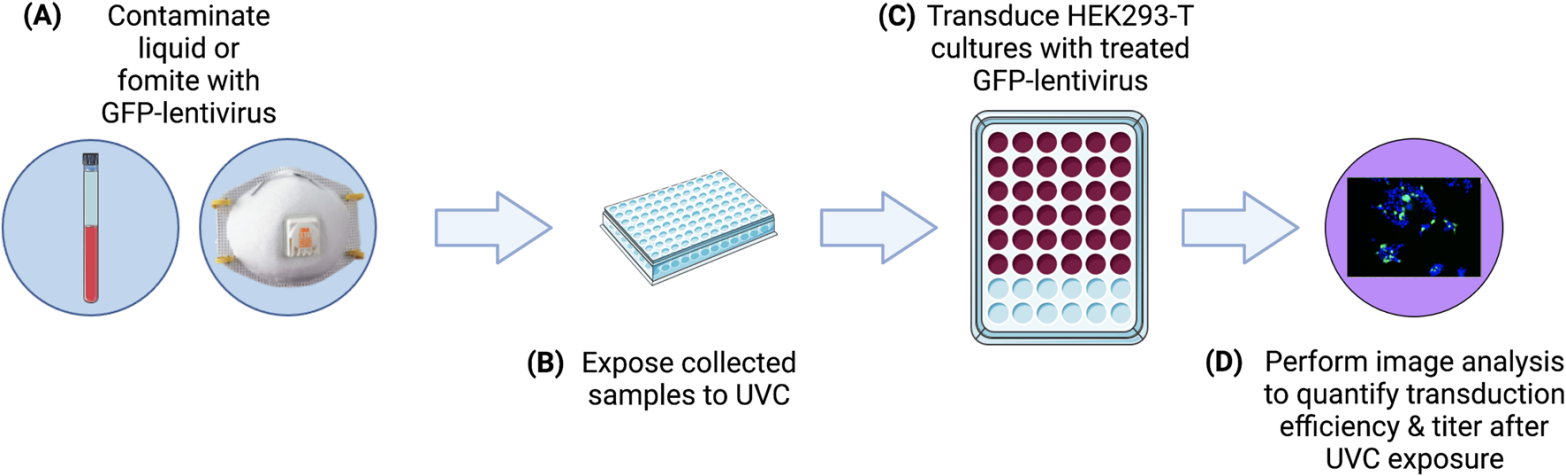
GFP-lentivirus experimental workflow. (**A**) Lentiviral samples are prepared either as a liquid medium or fomite. (**B**) A small representative portion of the sample is placed in a polystyrene 96-well plate and exposed to UVC light. (**C**) Treated samples are used to infect HEK293-T cells and incubated for two days. (**D**) A nuclear stain is added to facilitate cell counting using a high-content imaging device to determine a log reduction in virus infectivity after UVC irradiation.

Similar to SARS-CoV-2, lentiviruses are RNA viruses. We used a third-generation GFP encoded lentiviral vector as a model virus to study whether a medium surrounding the viruses impacts UVC disinfection. The experimental workflow is illustrated in Fig. 2. The lentivirus disinfection model is detailed in Fig. S2A–D. In this model, virus-infected cells express green fluorescent protein (GFP). To account for multiple possible infection events, a Poisson distribution was used to derive Eq. (2) for estimating the active viral concentration after UVC treatment. To validate this approach, we performed a standard curve experiment where we used tenfold dilutions to simulate viral reduction. Our model showed an exponential decay, matching the expected theoretical results (Fig. S2D).

**Figure 3:**
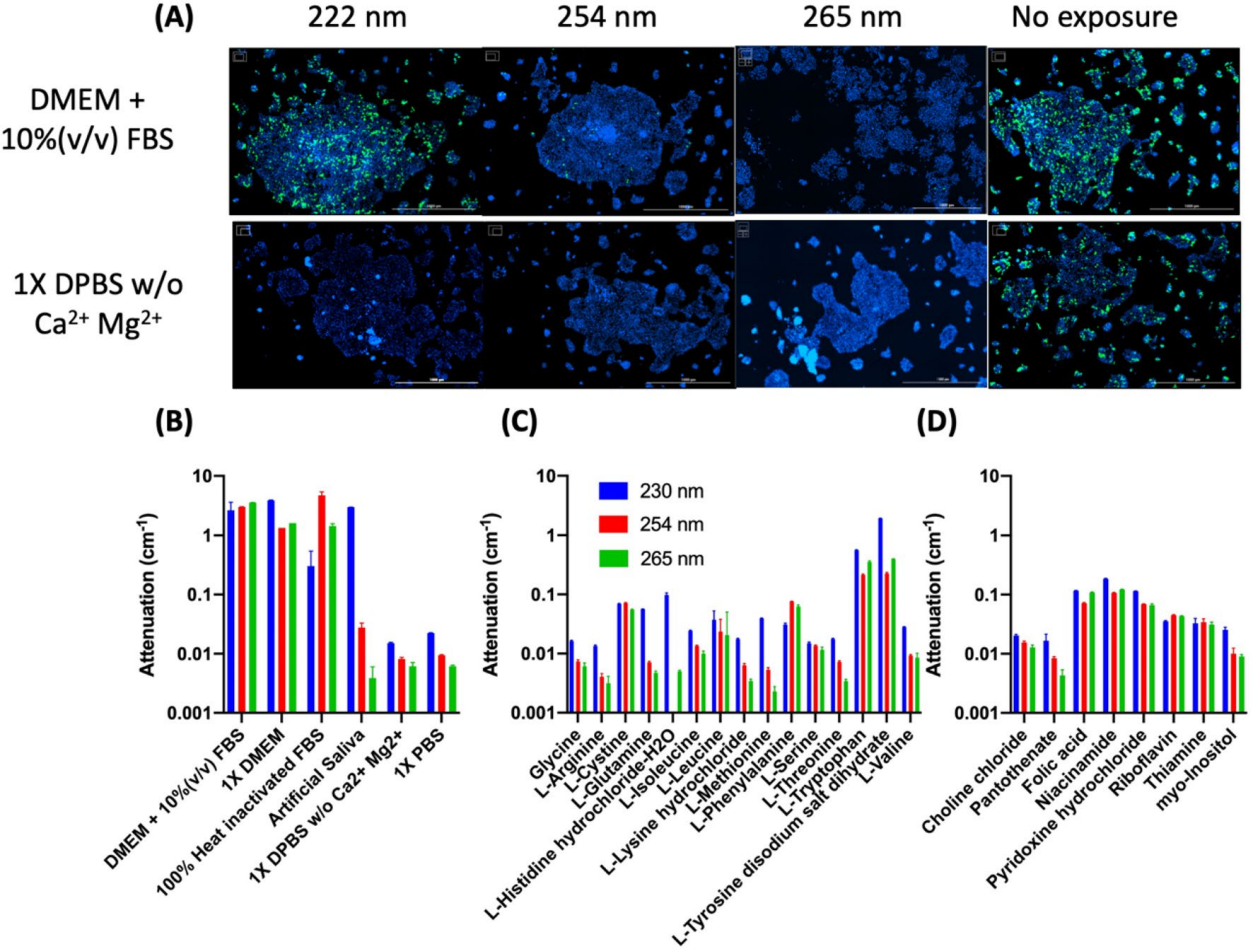
Effect of a medium on UVC virus disinfection. (**A**) Comparison of virus inactivation under different wavelengths (222 nm, 254 nm, and 265 nm) of UVC irradiation. A portion of 300 µL of lentivirus suspension in either DMEM + 10%(v/v) FBS (top panel) or DPBS without calcium or magnesium (bottom panel) was exposed to 4 J/cm^2^ of either 222, 254 or 265 nm UV light. Scale bar: 1000 µm. (**B**) Attenuation coefficient for suspension liquids used in UVC experiments. Attenuation coefficients for (**C**) amino acids and (**D**) vitamins in DMEM formulation.

Several reports provide widely varying inactivation UVC dosages for coronaviruses^34^. The effect of varying experimental conditions on the dose necessary to eradicate viruses using the aforementioned lentiviral model were investigated. Suspensions of 7 × 10^5^ TU/mL lentivirus in either Dulbecco’s phosphate-buffered saline (DPBS) without calcium and magnesium, or a complete cell culture medium (DMEM + 10%(v/v) FBS) were created. The suspensions were then exposed to 4 J/cm^2^ UVC light with different wavelengths (222 nm, 254 nm, and 265 nm), as shown in Fig. 3A. We observed no GFP expression in DPBS conditions for all tested wavelengths of UVC irradiation, indicating the complete inactivation of viruses under UVC. We detected GFP expression in all conditions for DMEM + 10%(v/v) FBS with visibly more expression in conditions exposed to 222 nm UVC light. An analysis of the components of DMEM and FBS revealed that DMEM contains several components that strongly attenuate UVC light. In general, the liquid suspensions used for the lentiviral experiments attenuated lower wavelengths more strongly than higher wavelength (Fig. 3B). Among typical amino acids used to formulate DMEM, L-tryptophan and L-tyrosine had attenuation coefficients above 0.1 cm^-1^ for 230, 254, and 265 nm (Fig. 3C). We provided absorbance measurements for 230 nm, as this is the lowest wavelength possible with our microplate reader. Among vitamins, niacinamide was the most strongly attenuating of 230, 254, and 265 nm (Fig. 3D). This discovery of vitamins and amino acids’ absorption of UVC is of particular interest, as many UV protection products can only block UVA and UVB light^52,53^. No product is available to block UVC. The discovery of potential UVC absorption reagents cold lead to the development UVC protection products.

**Figure 4:**
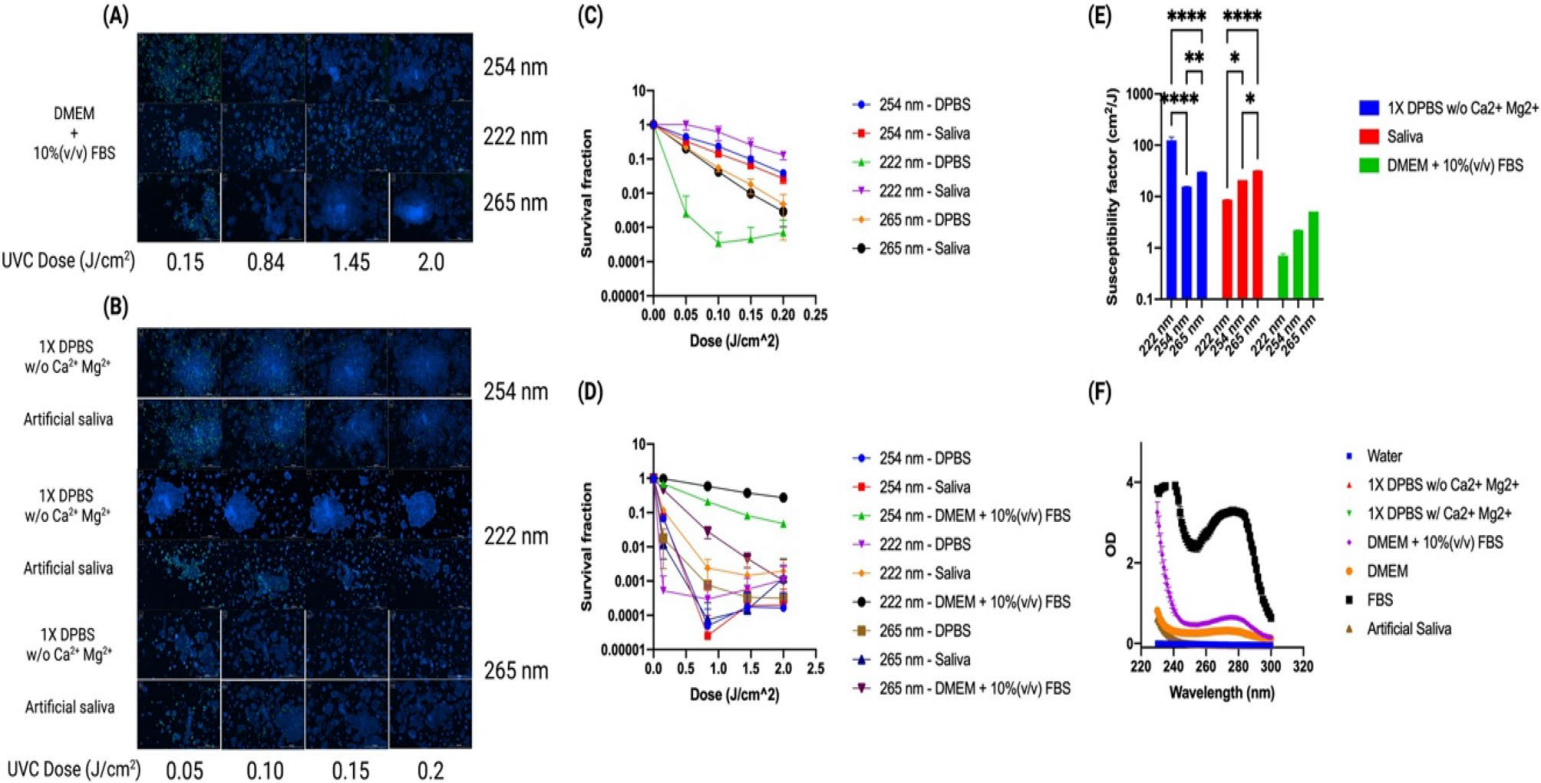
Lentiviral UVC susceptibility when being suspended in three different media. A portion of 300 µL of a lentivirus suspended in DPBS, an artificial saliva, or complete DMEM medium supplemented with 10%(v/v) FBS were exposed to various doses of 222, 254, or 265 nm UVC light. Lentivirus was exposed either to (**A**) 0.15–2 J/cm^2^ in DMEM + 10%(v/v) FBS or a (**B**) lower dose range of 0.05–0.2 J/cm^2^ before being used to infect HEK293-T cells. Cells were stained with a 1:2000 diluted Hoechst 33,342 at two days post infection. Scale bar: 1000 µm. Image analysis quantified the transduction efficiency of HEK293-T cultures for a (**C**) lower dose range and (**D**) the higher low dose range, and from these data (**E**) the susceptibility factors for lentivirus exposed to 222 nm, 254 nm, and 265 nm was calculated and compared. *p* value < 0.05. (**F**) Absorbance spectra of the three liquids showed DPBS absorbed both light sources negligibly across the 230–300 nm range. DMEM + 10%(v/v) FBS contains a secondary peak around 277 nm created by its components as well as the addition of the FBS itself. Error bars denote standard deviation. *p ≤ 0.05, **p ≤ 0.01, ***p ≤ 0.001, ****p ≤ 0.0001.

Next, we determined whether a decrease in GFP expression over a given range of irradiation dosages can be used as an index for the effectiveness of a given wavelength. In addition to the DMEM + 10%(v/v) FBS and DPBS, we added an artificial saliva solution as a suspension liquid. It is known that transmittable SARS-CoV-2 viruses are aerosolized in saliva^54^ droplets produced from a cough or sneeze^55^. As viruses in DPBS were completed inactivated by 4 J/cm^2^ UVC, we added a lower dose range for subsequent test. As shown in Fig. 4A–C, viruses were not inactivated completely under low dosages. Quantitative image analysis showed in the lower dose range that a greater than 3-log reduction of lentivirus in DPBS was achieved above a 0.1 J/cm^2^ incident dosage of 222 nm light (Fig. 4C). On the other hand, 222 nm was not able to achieve a 1-log reduction of lentivirus in DMEM + 10%(v/v) FBS at 2 J/cm^2^ (Fig. 4D). When a single-phase exponential decay curve is fitted, the average measured susceptibility factors for DPBS were 124.5, 15.67, and 30.23 cm^2^/J for 222, 254, and 265 nm, respectively. For saliva, the average measured susceptibility factors were 8.654, 20.99, and 31.96 cm^2^/J for 222, 254, and 265 nm, respectively. For a complete DMEM + 10%(v/v) FBS, the average measured susceptibility factors were 0.7027, 2.215, and 5.0980 cm^2^/J for 222, 254, and 265 nm, respectively (Fig. 4E). The absorbance spectra of all exposure liquids (DPBS, artificial saliva, and DMEM + 10%(v/v) FBS) were measured from 230 to 300 nm. All liquids tended to attenuate more light the lower the wavelength. However, DMEM + 10%(v/v) FBS had a secondary absorbance peak at 277 nm (Fig. 4F). Further absorbance spectral analysis reveals that both the components in the DMEM and the FBS contribute to the secondary peak, with the FBS contributing the most.

#### Effect of medium composition and liquid depth on virus UVC susceptibility

**Figure 5:**
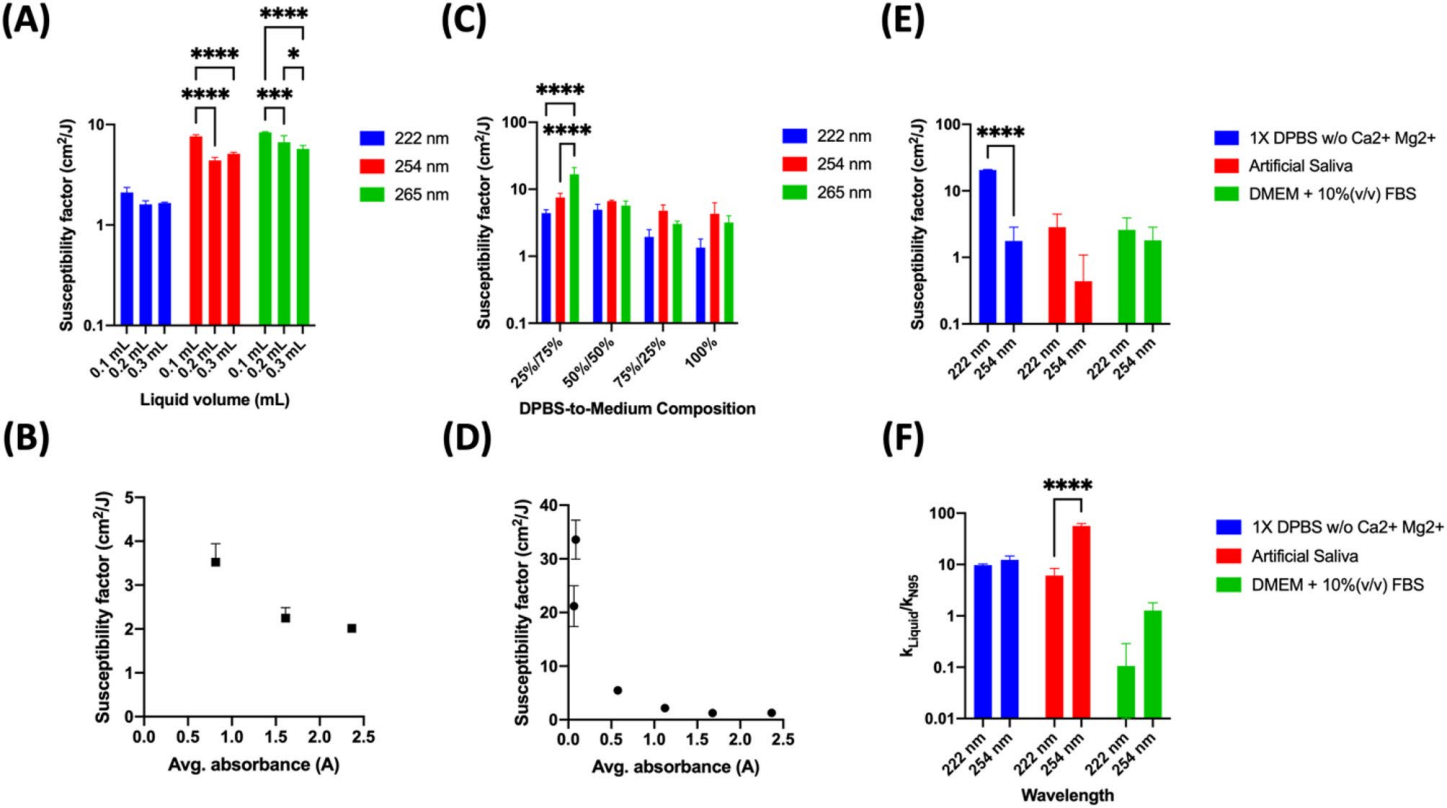
Environmental effect on virus UVC susceptibility. (**A**) Viruses suspended in DMEM + 10%(v/v) FBS with different liquid depths created by adding different volumes (0.1, 0.2, and 0.3 mL) of virus suspension to a 96-well plate. Viruses were exposed to various does of 222 nm, 254 nm, or 265 nm UVC light from which susceptibility factors were calculated. *p* values < 0.05. (**B**) The correlation of virus susceptibility to liquid absorbance of UVC light at 254 nm. The 254 nm viral susceptibilities were negatively correlated to the measured liquid absorbance at the three liquid heights. (**C**) Mixtures containing different percentages of DMEM + 10%(v/v) FBS (25, 50, 75, and 100%) to DPBS were exposed to 222 nm, 254 nm, and 265 nm UVC light. (**D**) The 254 nm viral susceptibility factors were negatively correlated to the measured absorbances of the different mixtures. (**E**) N95 mask cut-out samples were contaminated with 10 µL of a concentrated viral suspension and then irradiated to 254 nm and 222 nm at various doses. Virus was significantly more susceptible to 222 nm in DPBS. (**F**) N95 masks decreased the susceptibility of virus tenfold when compared to a pure liquid environment. Error bars denote standard deviation. *p ≤ 0.05, **p ≤ 0.01, ***p ≤ 0.001, ****p ≤ 0.0001.

A comprehensive review of coronavirus inactivation studies suggested that many of the reported UVC viral inactivation doses were high because the light was attenuated highly by the suspension medium^34^. Absorbance is a function of both the material and the light pathlength, so we decided to characterize whether both would show a correlation between the susceptibility factor of the virus in suspension. We suspended the viruses in different volumes of the DMEM + 10%(v/v) FBS to create a range of liquid depth from 0.1 to 0.3 mL (3.1 mm to 9.4 mm). As shown in Fig. 5A, we observed that the viral susceptibility was higher with a smaller volume of liquid, resulting a shorter optical pathlength. In general, viral susceptibility factors were higher for 0.1 mL than 0.3 mL for all three wavelengths. Moreover, we noted an inverse correlation between the absorbance and susceptibility factors at 254 nm UVC irradiation (Fig. 5B). We then created viral suspensions using 25%, 50%, and 75% DMEM + 10%(v/v) FBS mixed with DPBS and repeated the experiments. Raising the percentages of DMEM + 10%(v/v) FBS in the mixture increased the overall absorbance across the 230–300 nm wavelength range, but also created a local peak around the 270–280 nm range, as described earlier. As with the liquid height study, viral susceptibility factors for the three wavelengths decreased as the percentage of DMEM + 10%(v/v) FBS increased (Fig. 5C). We observed an inverse non-linear correlation between the two variables (Fig. 5D). Measured susceptibilities were highly variable when the viral experiments were performed in suspension liquids with low absorbances, but plateaued for absorbances above 1.5. We only performed this characterization for 254 nm susceptibilities since our plate reader could only measure as low as 230 nm, and absorbance measurements of the suspension liquids used in our study frequently produced overflow values around 230 nm.

### Virus inactivation on N95 mask cut-out samples

N95 respirators are essential components of personal protective equipment (PPE) that can protect the wearer from external airborne pathogens as well as minimizing the introduction of contagious pathogens into the air from respiratory exhalent. There have been increasing efforts to evaluate different methods to sterilize them for reuse during the COVID pandemic when PPE shortage threated frontline healthcare workers. We used our lentiviral model to ascertain whether we could measure the viral susceptibility in the environment of the N95 masks while also considering the effect of the suspension liquid. We kept the amount of active virus the same as in our experiments in liquid suspensions of 300 µL but condensed the delivery volume to 10 µL to be applied to a 7-mm diameter cut-out N95 mask samples. As a control, we applied the same virus suspension to N95 mask cut-outs but did not expose them to a UVC light. The viral reduction calculated therefore was based on this control. Viral susceptibility to 222 nm was much higher than to 254 nm in the N95 mask environment for the case of DPBS conditions (Fig. 5E). Although we calculated slightly increased 222 nm susceptibilities for saliva and complete cell culture medium, they were not significantly higher than those of 254 nm. Virus suspensions in DPBS were 10 × more susceptible in the purely liquid environments than on the N95 mask for both 222 nm and 254 nm, but we noted for saliva and cell culture medium that the different wavelengths performed differently (Fig. 5F). The effectiveness of 254 nm irradiation was reduced by 56-fold by the N95 mask but for 222 nm only sixfold. For lentivirus in DPBS, the N95 mask reduced the effectiveness for 254 nm and 222 nm similarly by tenfold. For lentivirus in DMEM + 10%(v/v) FBS, the N95 mask did not reduce the effectiveness.

### Evaluation of UVC light safety and implementation

**Figure 6:**
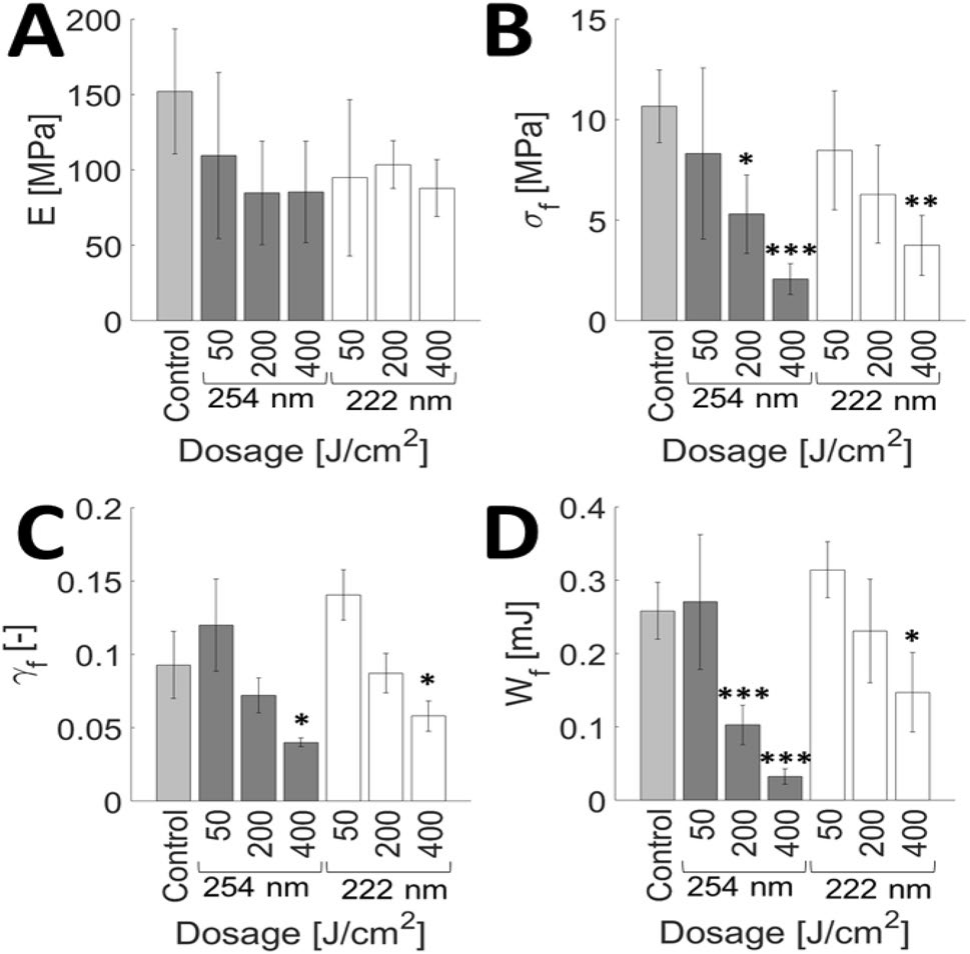
UVC light induces changes in the mechanical properties of SC equilibrated to 25% RH. Average (**A**) elastic modulus, $E$, (**B**) fracture stress, $\sigma_{f}$, (**C**) fracture strain, $\gamma_{f}$, and (**D**) work of fracture, $W_{f}$ for unirradiated controls (Control; light grey), 254 nm irradiated samples (dosage range: 50–400 J/cm^2^; dark grey), and 222 nm irradiated samples (dosage range: 50–400 J/cm^2^; white). Bars denote average values of $4 \le n \le 5$ individual sample measurements for each range and dosage condition. Error bars denote standard deviations. Confidence intervals are indicated by *p ≤ 0.05, **p ≤ 0.01, ***p ≤ 0.001.

**Figure 7:**
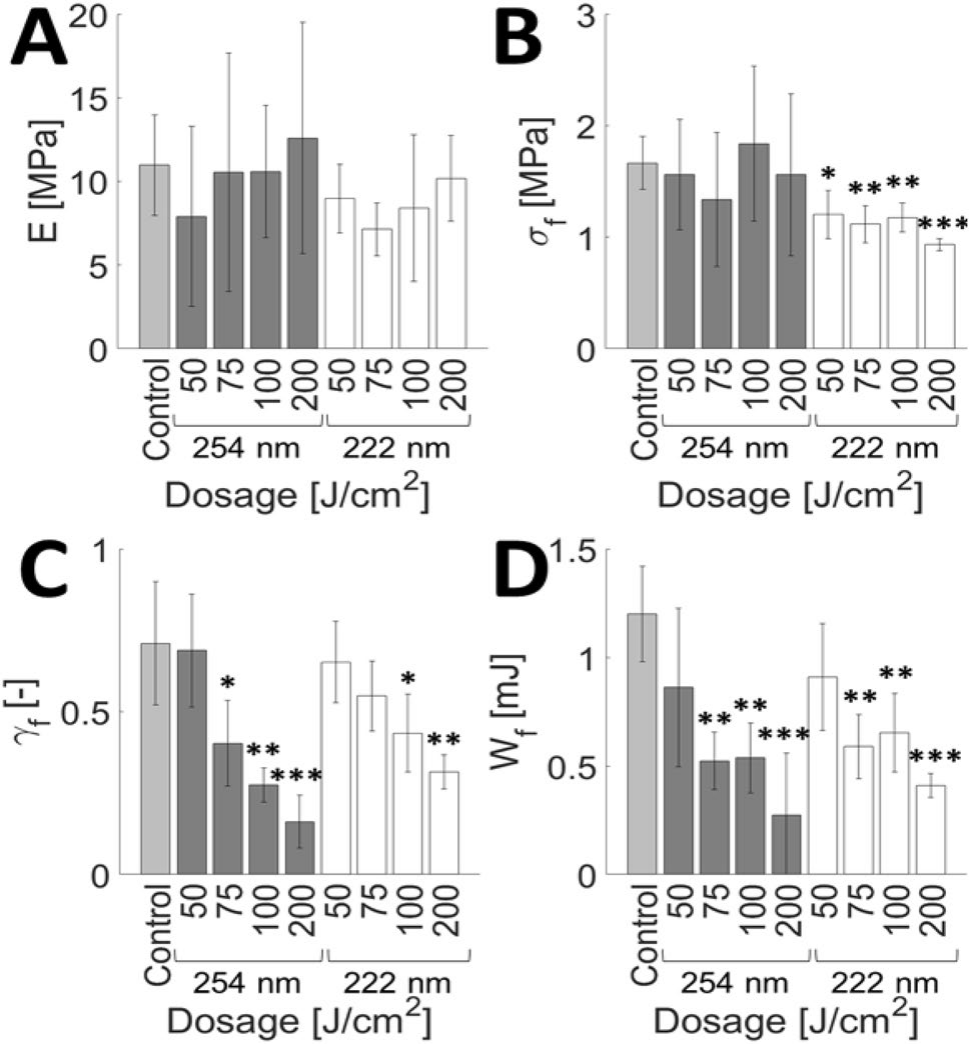
UVC light induces changes in the mechanical properties of SC equilibrated to 100% RH. Average (**A**) elastic modulus, ${ }E$, (**B**) fracture stress, $\sigma_{f}$, (**C**) fracture strain, $\gamma_{f}$, and (**D**) work of fracture, $W_{f}$ for unirradiated controls (Control; light grey), 254 nm irradiated samples (dosage range: 50–200 J/cm^2^; dark grey), and 222 nm irradiated samples (dosage range: 50–200 J/cm^2^; white). Bars denote average values of $4 \le n \le 5$ individual sample measurements for each range and dosage condition. Error bars denote standard deviations. Confidence intervals are indicated by *p ≤ 0.05, **p ≤ 0.01, ***p ≤ 0.001.

While UVC can be an effective antigermicidal tool to reduce the rate of transmission in congregate settings, safety risks must be considered before implementing such UV light sources. Promising research in 222 nm UVC light has demonstrated that it may be safer than 254 nm as it does not cause DNA damage in a human skin, nor does it cause cytotoxic damage to mammalian skin^20,22,37^. We intended to determine what effect chronic exposure to these two UV light sources may cause to the mechanical properties of human stratum corneum (SC), the outermost barrier of skin that protects underlying living tissue from external insults. Figure 6A–D show the average elastic modulus,$E$, fracture stress, $\sigma_{f}$, fracture strain,$\gamma_{f}$, and work of fracture $, W_{f}$, of SC after exposure to different incident dosages of 222 and 254 nm light, followed by equilibration for 24 h to 25% relative humidity (RH) prior to mechanical testing. Figure 6A shows that there is no significant change in the elastic modulus of any irradiated samples relative to the control tissue. However, Fig. 6B, D highlight that fracture stress and the work of fracture, respectively, decrease at dosages above 400 J/cm^2^ for 222 nm and above 200 J/cm^2^ for 254 nm relative to the control tissue. Figure 6C shows that for both 222 nm and 254 nm irradiation, the fracture strain decreases above 400 J/cm^2^ relative to the control tissue. Figure 7A–D show the average elastic modulus,$E$, fracture stress, $\sigma_{f}$, fracture strain,$\gamma_{f}$, and work of fracture $, W_{f}$, of SC after irradiation with different dosages of 254 and 222 nm light, followed by equilibration for 24 h to 100% RH prior to mechanical testing. Similar to Figs. 6A, 7A shows that there is no significant change in the elastic modulus at 222 or 254 nm irradiated samples relative to control tissue. Figure 7B highlights that while fracture stress of 254 nm irradiated samples does not significantly change for any dosage, as compared to controls, it does notably decrease for 222 nm irradiated samples at dosages as low as 50 J/cm^2^. Figure 7C shows that fracture strain decreases at dosages above 100 J/cm^2^ for 222 nm and above 75 J/cm^2^ for 254 nm. The work of fracture in Fig. 7D decreases at dosages above 75 J/cm^2^ for both 222 and 254 nm.

**Figure 8:**
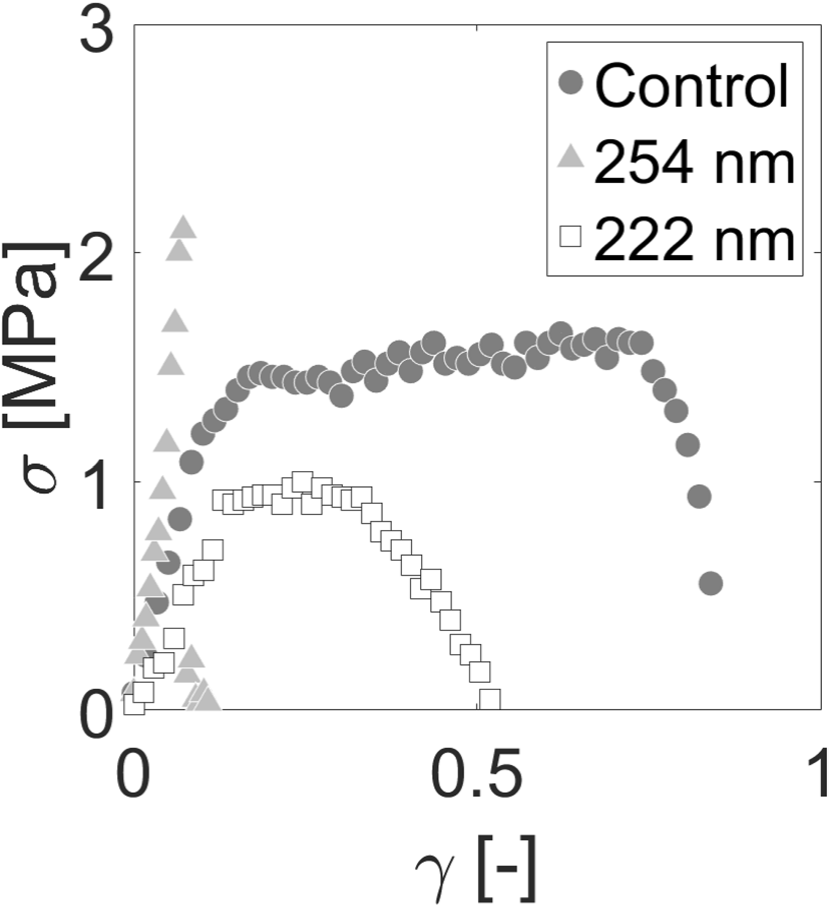
Loss of SC plastic deformability with UVC irradiation. Representative stress–strain plots of an unirradiated SC sample (dark grey circle), a sample irradiated with 200 J/cm^2^ of incident 254 nm light (light grey triangle) and a sample irradiated with 200 J/cm^2^ of incident 222 nm light (white square). All samples are equilibrated to 100% RH.

Degradation trends for SC samples irradiated with 254 nm at both high and low humidity conditions seems to agree with our previously published data^38^. It would also seem that 222 nm light is just as harmful as 254 nm light to the mechanical integrity of SC. Moreover, the amount of energy needed to cause significant damage to the SC tissue is reduced for high humidity conditions (75 J/cm^2^ for 222 and 254 nm light) than at low humidity conditions (400 J/cm^2^ for 222 nm and 200 J/cm^2^ for 254 nm light). This can be attributed to a significant lack of plastic deformability at high humidity conditions, as illustrated in Fig. 8.

## Discussion

We report a SARS-CoV-2 UVC susceptibility of 1,239 cm^2^/J and 281 cm^2^/J in PBS and EMEM + 2%(v/v) FBS, respectively. Due to the discrepancy of observed susceptibilities, we developed a GFP lentivirus-based quantitative model to ascertain the effectiveness of different UVC wavelengths. We were able to accurately determine up to a 4-log reduction using a standard curve with the virus UVC dose titration platform that we developed (Fig. S2E). The platform using the GFP lentivirus exhibited a dose-dependent inactivation when exposed to UVC light (Fig. S3). The irradiation from different light sources was mapped to develop an irradiance-dosage model for determining inactivation dosages (Fig. S3). The viral suspension formula used by many groups has been employed to experimentally determine the UVC inactivation dosages for many viral pathogens^56–61^. We showed that these doses can be overestimated if the properties of the liquid suspensions are not taken into account. As we discovered in this study, absorbance and susceptibility are inversely correlated with each other, whether due to the material or change in the optical pathlength. As such, inactivation studies examining aerosolized viral particles need to account for the optical pathlengths through droplets of polydisperse radii. This would likely explain why recommended inactivation doses for aerosolized particles are notably smaller than the dosages determined by the liquid suspension methodology^13,62^. Inactivation studies often employ liquids commonly present in laboratories. Our work suggests that the adoption of more relevant liquids such as saliva may give more physiologically relevant and meaningful inactivation dosages. Liquids that minimally attenuate a particular wavelength should be used with caution for viral inactivation studies as they may underestimate required inactivation dosages in more relevant situations.

Due to reduced attenuation, we observed a higher susceptibility of the virus to 222 nm UVC light in PBS as compared to 254 and 265 nm light. This suggests that 222 nm is more effective than 254 nm for viral inactivation when UVC absorbing elements are absent. This was observed in both pure liquid suspensions and on N95 masks. The finding is consistent with 222 nm containing more photonic energy than 254 nm light. However, in highly UVC-absorbing liquids such as cell culture medium with serum, 265 nm was the more effective wavelength. We observed that 222 nm light was more potentially more effective than 254 nm light for disinfecting N95 mask cut-outs However, more testing is necessary to characterize how the mask functional and mechanical properties change due to UVC exposure.

The generation of ozone by far UV sources is a potential factor that we did not consider when examining the results of our experiments using the 222 nm light source. However, work by Welch et al. suggested that ozone generation in air is less than 0.005 ppm^63^.

Other groups have reported 260–270 nm as the most effective wavelength when compared to higher wavelengths (280–300 nm)^64,65^, or to the traditional 254 nm^66^. 255 nm UV LEDs had a higher germicidal efficiency when compared to 280 nm and 350 nm^67^. When 170, 222 and 254 nm were compared to disinfect *Bacillus subtilis* spores, 222 nm was determined to be the most effective. While study reported in this work supports these effective UVC ranges, our study highlights the necessity to consider carefully the environmental factors where disinfection is necessary. The comparison of these wavelengths also suggested that the UVC disinfection devices using multiple wavelengths may be the best strategy to maximize germicidal efficiency in all environments and three key UV-mediated microbial inactivation mechanisms have been proposed^68^: (i) protein damage and DNA repair suppression by 190–254 nm, (ii) direct and indirect DNA damage by 250–320 nm, (iii) and generation of reactive oxygen species by endogenous photoinitiators by 300–405 nm. Our results supported the hypothesis that 222 nm is the most effective disinfecting wavelength, but only in conditions where lower UVC wavelengths are not highly absorbed. The generation of damaging reactive oxygen species by higher UV wavelength led us to hypothesize that a dual wavelength approach of 222 nm plus broad spectrum UV source centered around 265 nm would optimally engage all three mechanisms and thus be a more robust disinfection system than any single wavelength.

The safety of different UV light sources must be weighed with their application. While we did not observe significant decreases in the elastic modulus of skin exposed to either 254 nm and 222 nm, there were decreases to the fracture stress, fracture strain, and work of fracture. Stratum corneum equilibrated to 100% relative humidity showed mechanical degradation at dosages of 50 J/cm^2^, while stratum corneum equilibrated to 25% relative humidity required doses exceeding 200 J/cm^2^, relative to controls. It has been reported that less than 5% of far UV light reaches the center of cells of a typical diameter of 10 µm^69^. The stratum corneum comprises terminally differentiated corneocyctes and serves as a protective barrier against far UV light. Previous reports have focused on looking for cytotoxic and DNA damage^20,22^ to skin but our findings here suggest an indirect mechanism by which chronic far UV exposure could lead to the degradation of the integrity of skin, which could leave it more susceptible to infection. It is therefore essential that if far UV technology is to be implemented in public settings, it must be done so in such a way that delivers the minimally necessary inactivation dose required, thereby minimizing public exposure.

## Supplementary Information

Supplementary Information.

## Data Availability

All datasets from this study are available from the corresponding author upon reasonable request.
