# Supplementary material for: Systematic evaluating and modeling of SARS-CoV-2 UVC disinfection
Source: Sci Rep. 2022 Apr 7;12:5869. doi: 10.1038/s41598-022-09930-2 (PMC8988105; doi:10.1038/s41598-022-09930-2)
Supplement: Supplementary file 1 — Supplementary Information. [file 41598_2022_9930_MOESM1_ESM.docx]

**Supplementary Figures**.


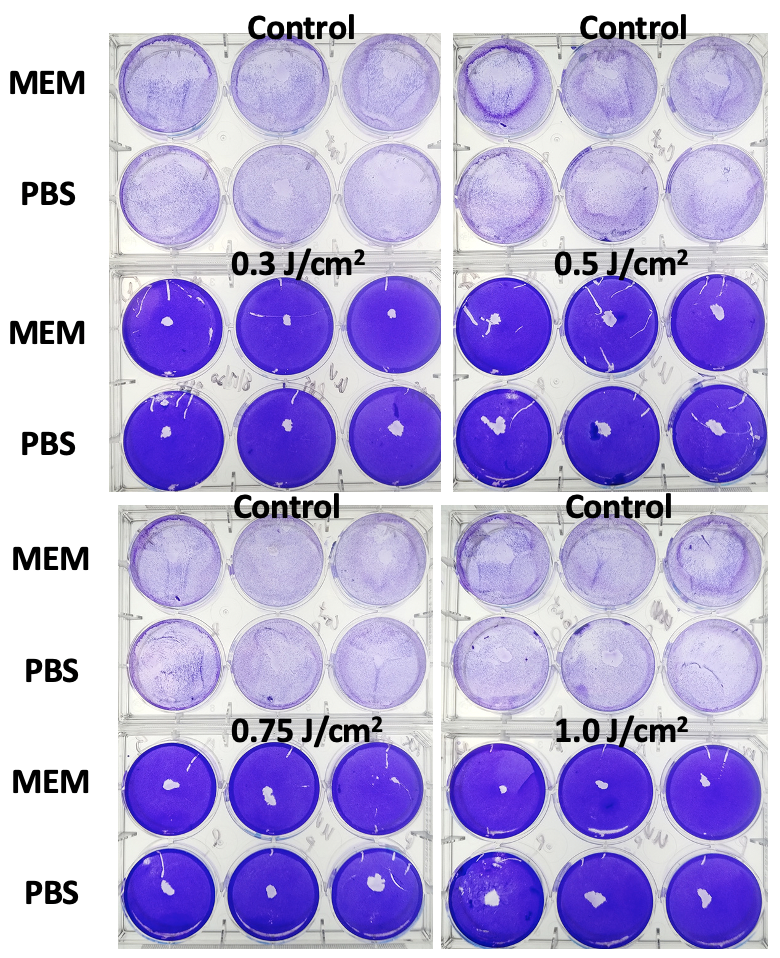


**Supplementary Figure 1**. **Effect of liquid on SARS-CoV-2 inactivation**. A UVC-254 nm lamp was used to irradiate a 96-well plate containing 300 µL of a 10^6^ pfu/mL SARS-CoV-2 suspension. Dose times were calculated based on the measured irradiance and the desired exposure dose for each condition. All conditions were frozen and the plaque-forming assay was run a few days later. Controls were treated exactly the same, but did not receive any UV light exposure. For a range between 0.3 – 1 J/cm^2^, no difference between conditions was observed and no cytopathic effect was detected in cultures. All conditions were performed in triplicates.


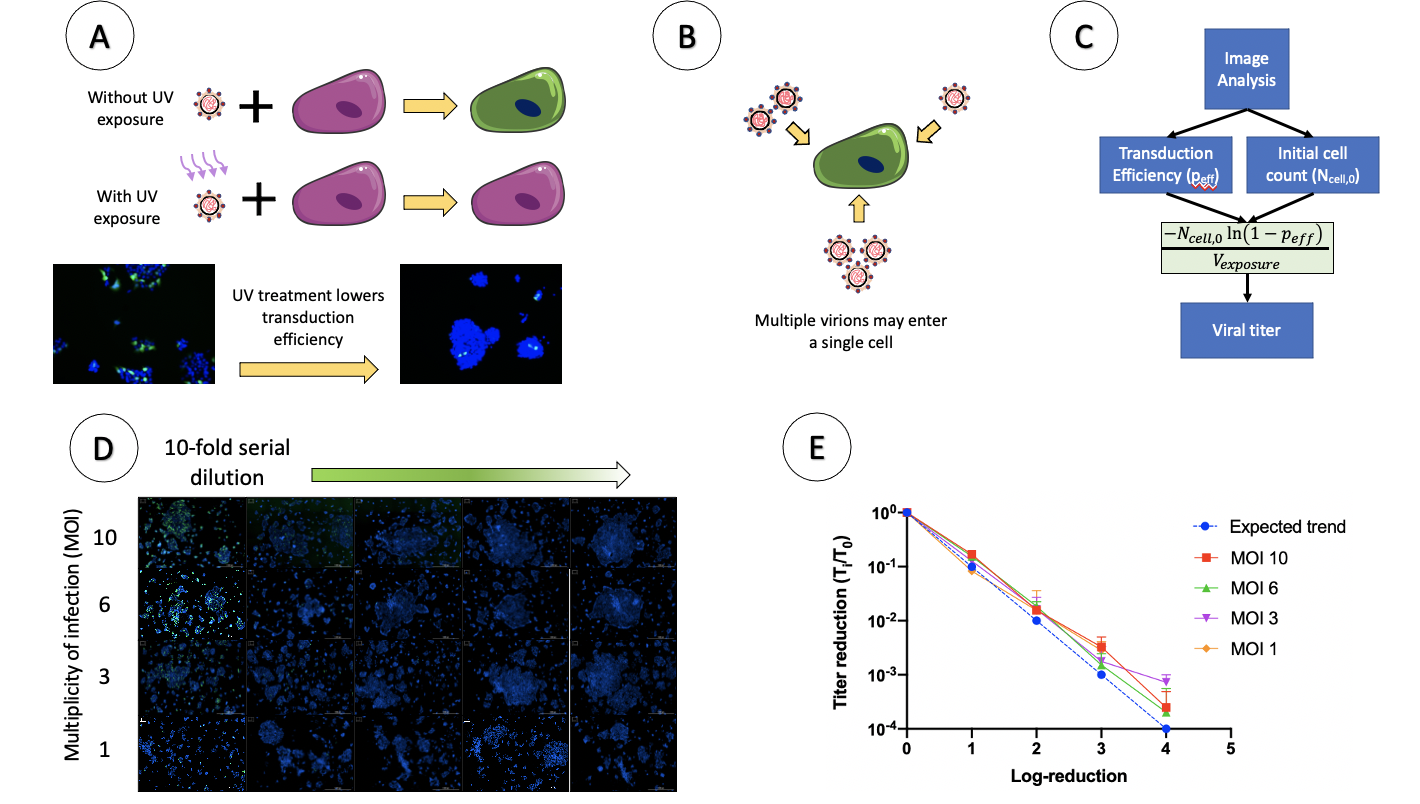


**Supplementary Figure 2. Establishment of a GFP-lentiviral disinfection model**. A) The transduction of HEK 293-T cells by GFP-lentivirus was adopted as a model of disinfection. HEK293-T GFP expression was used as a visual indication that a lentivirus particle had infected a cell. B-C) Our model was derived from a Poisson distribution in order to account for multiple infection events. D) 10-fold serial dilutions of various starting multiplicities of infection (MOI) mimicked viral reduction that could be observed in an experiment. E) The image data from the serial dilution experiment closely matched the theoretically expected result.


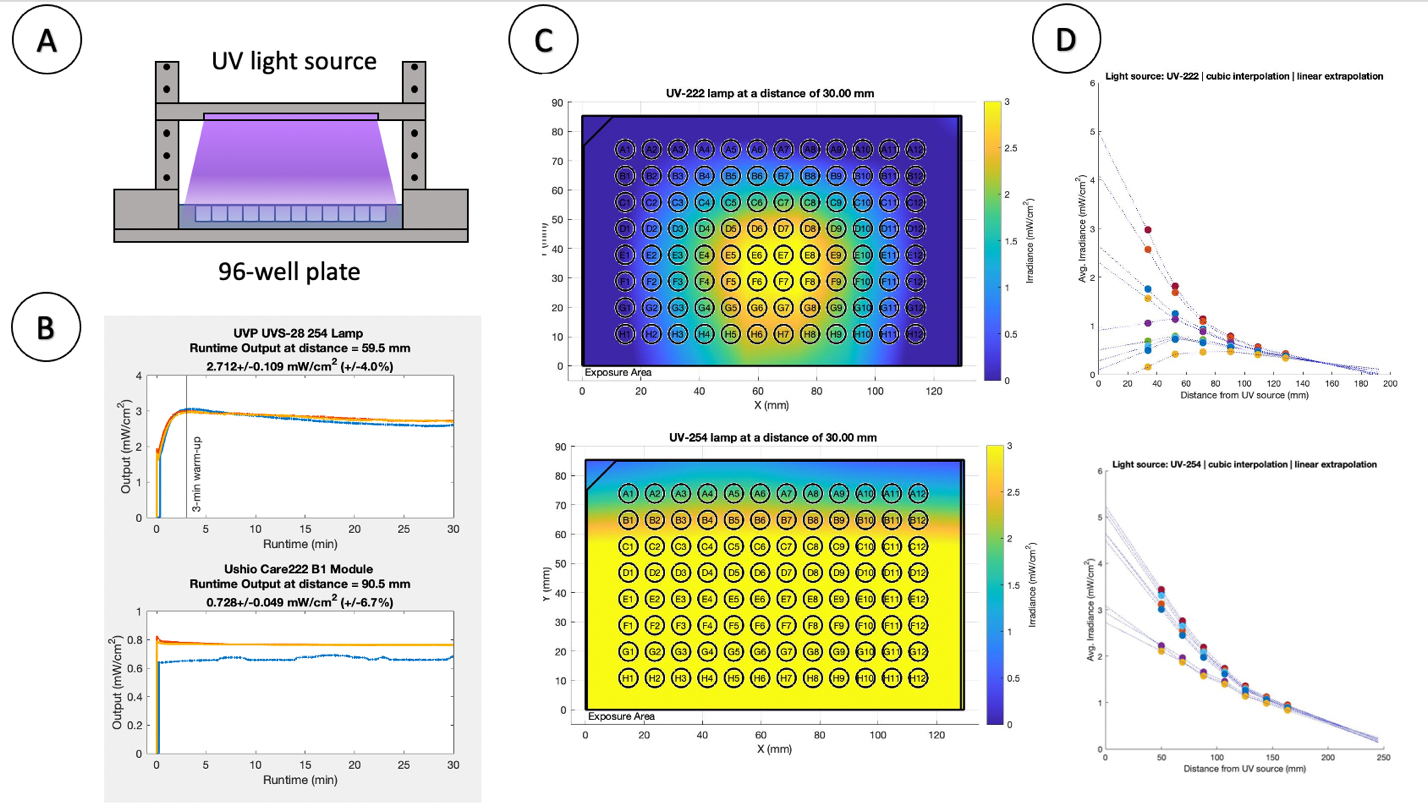


**Supplementary Figure 3**. **UV exposure setup**. A) An aluminum scaffold was built to elevate a UV light source over a securely held 96-well plate. The UV light source was either i) 254 nm low-pressure mercury lamp (UVS-26, Analytik-Jena), ii) 222 nm excimer lamp (Ushio), or a prototype 265 nm LED strip (Crystal IS) (data not shown). B) The 254 nm lamp required at least a 3 min warm-up time and output 2.7 mW/cm^2^ at 59.5 mm. The 222 nm lamp had no warm-up time and output 0.7 mW/cm^2^. Measurements were made with a UV light meter (International Light Technologies, Peabody, MA) .B) UV light sources were characterized for their stability over time and a UV irradiance map (mW/cm^2^) were generated for the 96-well hold space. C) Viral suspensions or fomites (such as N95-well respirator mask cut-out samples contaminated with viral suspensions) were placed into the wells of a 96-well plate for UV exposure. The UV meter was used to determine irradiance spatial distribution at various height settings for the exposure setup. Full spatial maps were created from data interpolation/extrapolation at a height of 3 cm away. D) Data interpolation/extrapolation. Measurements were made using a ILT2400 with a SED220/W calibrated sensor (International Light Technologies, MA). The exposure area was broken up into 36 evenly spaced grid point. Points needed to be interpolated/extrapolated for well irradiance calculations, which fell outside of the measured grid points. MATLAB’s griddedInterpolant function was used for this purpose. A cubic interpolator and linear extrapolator were used for all UV light sources.
